# Sleep status of older adults with sleep apnoea syndrome may vary by body mass index

**DOI:** 10.3389/fragi.2024.1331448

**Published:** 2024-05-01

**Authors:** Yuji Tanaka, Naana Baba-Mori, Takaaki Yonaga, Kazuki Mochizuki, Satoshi Igarashi, Takashi Ando, Takashi Kohda, Yasumi Ito, Kenzo Soejima, Daiju Sakurai

**Affiliations:** ^1^ New Industry Creation Hatchery Center, Tohoku University, Sendai, Japan; ^2^ Department of Advanced Biomedical Research, Faculty of Medicine, University of Yamanashi, Yamanashi, Japan; ^3^ Department of Otorhinolaryngology, Head and Neck Surgery, Faculty of Medicine, University of Yamanashi, Yamanashi, Japan; ^4^ Laboratory of Food and Nutritional Sciences, Department of Local Produce and Food Sciences, Faculty of Life and Environmental Sciences, University of Yamanashi, Yamanashi, Japan; ^5^ Department of Orthopaedic Surgery, Faculty of Medicine, University of Yamanashi, Yamanashi, Japan; ^6^ Laboratory of Embryology and Genomics, Faculty of Life and Environmental Sciences, University of Yamanashi, Yamanashi, Japan; ^7^ Faculty of Engineering, University of Yamanashi, Yamanashi, Japan; ^8^ Department of Respiratory Medicine, Faculty of Medicine, University of Yamanashi, Yamanashi, Japan

**Keywords:** apnoea-hypopnea index, apnoea syndromes, body mass index, sleep stage, older adults, ageing

## Abstract

Obesity and ageing are the most important risk factors for sleep apnoea syndrome (SAS); however, the role of body mass index (BMI) on sleep status in healthy older adults is unclear. To explore sleep parameters according to BMI among active older adults, we cross-sectionally examined the relationship between sleep-related parameters and BMI in 32 Japanese adults aged from 83 to 95 years without long-term care who were unaware of having SAS. Correlation and linear regression analyses were performed. Moderate or severe SAS prevalence was high in both those with low (68.8%) and high (68.8%) BMI. A higher increase in apnoea-hypopnoea index (AHI) was negatively correlated with sleep depth in the high-BMI group. In the low-BMI group, the number of awakenings and age were positively correlated with AHI. Older adults may have SAS regardless of their BMI, and the sleep status of patients with SAS may vary by BMI.

## 1 Introduction

Sleep apnoea syndrome (SAS), defined as moderate or severe SAS (Apnonea-hypopnea index [AHI] ≥15/h) affects approximately 2%–50% of the middle-aged adult population in the U.S. and Australia (Young et al., 1993; Baldwin et al., 2001; Banno and Kryger, 2007; Peppard et al., 2013; Heinzer et al., 2016; McArdle et al., 2022), and the prevalence among patients with stroke exceeds 60% (Mohsenin and Valor, 1995; Bassetti and Aldrich, 1999; Parra et al., 2000). Well-documented risk factors for SAS include ageing (Young et al., 2002; Jordan et al., 2014; Matsumoto et al., 2020), obesity (Phillips et al., 1999; Newman et al., 2005; Yaggi et al., 2005; Hudgel et al., 2012; Lam et al., 2012; Fan et al., 2019), snoring (Martínez-García et al., 2012; Heinzer et al., 2015), high cholesterol levels (Nadeem et al., 2014), high red blood cell counts (Feliciano et al., 2017), smoking (Yaggi et al., 2005; Martínez-García et al., 2012; Heinzer et al., 2015), and male sex (Martínez-García et al., 2012). Of these, old age and obesity are major factors with increasing global prevalence, making SAS an important future concern. A weight gain of ≥10 kg is associated with a 5-fold risk of moderate or severe SAS in men and a 2.5-fold risk of moderate or severe SAS in women (Newman et al., 2005). We recently examined the prevalence of SAS in 32 relatively healthy older adults (average age, 87 years) in Yamanashi Prefecture who were not certified for long-term care, wherein 68.8% were found to have moderate or severe SAS (AHI ≥15/h), all of whom were unaware that they had SAS (Tanaka et al., 2022), suggesting a lack of awareness of this condition among older adults.

Although many studies have addressed SAS, particularly in populations aged <60 years, it remains unclear how the combination of ageing and obesity affects the prevalence and severity of SAS in older age groups. An analysis of 30 participants in a longitudinal study in San Francisco (baseline age, 57.7 years; mean age at last follow-up, 82.0 years) reported that sleep apnoea worsens with age, regardless of weight gain or loss (Bliwise et al., 2010). Additionally, a study conducted in Nagahama, Japan (Matsumoto et al., 2020) showed that the incidence of moderate or severe SAS exceeded 10% in adults aged >70 years with low body mass index (BMI). In our small multifaceted exploratory study of Japanese adults aged >80 years, muscle weakness and poor exercise habits were the main factors associated with SAS severity, with BMI not identified as a significant SAS-related factor (Tanaka et al., 2022). These findings suggest that some older adults develop SAS, although they have a low BMI. SAS-related factors in older adults with low BMI have not yet been investigated, and it is unclear whether differences in characteristics and pathophysiology exist between older adults with high and low BMI. To the best of our knowledge, no studies have investigated how the combination of ageing and BMI affects sleep quality.

This study aimed to determine how BMI affects sleep in older adults. Specifically, we aimed to explore sleep parameters (sleep depth, body position, number of awakenings during sleep, and AHI) according to BMI status among a cohort of adults aged >80 years (Tanaka et al., 2022) who were relatively healthy and did not require long-term care. These will contribute to the inference of risk factors and mechanisms of SAS in older adults and will serve as a catalyst for more detailed validation studies and biomarker searches to communicate risk to older adults.

## 2 Materials and methods

### 2.1 Study design, setting, and participants

In 2019, we invited 104 people from the Yamanashi Healthy-Active Life Expectancy study cohort (Kondo et al., 2008) to participate in the Yamanashi Healthy active long-living older people Biobank for healthy ageing biosciences and an intermediate questionnaire survey (Tanaka et al., 2022). We included participants who 1) did not require long-term care, 2) provided informed consent for the survey and SAS measurements, and 3) did not give the researchers the impression that they had difficulty participating in the study (e.g., a participant with poor understanding of the sleep measurement kit despite being told how to use it or who could not wear the sensor). All measurements, including the sleep apnoea test, were performed between January and December 2020. We excluded those for whom the SAS test could not be adequately performed (e.g., where measurement errors occurred).

### 2.2 Ethical considerations

This study was approved by the Ethics Committee of University of Yamanashi School of Medicine (approval number: 2096; approval date: December 2019). The study complied with the Declaration of Helsinki, the Japanese Ethical Guidelines for Medical Research Involving Human Subjects, Act on the Protection of Personal Information, and Ethical Guidelines for Human Genome and Genetic Analysis Research. We obtained written informed consent from the participants after explaining the study verbally and in writing.

### 2.3 Measurements

#### 2.3.1 Physical measurements

Participants’ weight multi-frequency segmental body composition analyzer (Tanita MC-780A-N; Tanita Corp., Tokyo, Japan) (Yamada et al., 2017). Weight was measured by subtracting the weight of clothing (1 kg in January and February to account for heavier winter clothing and 0.5 kg from March to December). Height was measured using a stadiometer (Height Measurement HM 200P; Charder Electronic Co., Ltd., Taichung City, Taiwan). Systolic blood pressure, diastolic blood pressure, and pulse rate were measured using a sphygmomanometer (Terumo ES-W300ZZ; Terumo Corp., Tokyo, Japan).

#### 2.3.2 Lifestyle and medical history questionnaire

Each participant completed a questionnaire on lifestyle habits and medical history, which included questions about alcohol consumption, smoking, and history of SAS.

#### 2.3.3 Sleep apnoea test

The AHI was measured using a portable monitoring device (WatchPAT 200; Itamar Medical, Caesarea, Israel) as recommended in the American Academy of Sleep Medicine guidelines for obstructive SAS (Kapur et al., 2017). The gold-standard polysomnography (PSG) is a high-burden test for participants unaware of SAS. Because of the older age of the participants, we also avoided using a device that required the wearing of a unit to measure airflow through the nose and decided to use the WatchPAT 200, which is certified as a medical device as a simple testing device. Peripheral arterial tone signal measures the arterial pulsatile volume changes of the finger that are regulated by α-adrenergic innervation of the smooth muscles of the vasculature of the finger; thus, it reflects sympathetic nervous system activity. This augmentation in sympathetic activity accompanies increased heart rate and desaturation at the termination of respiratory events. The WatchPAT recorder is based predominantly on recordings of peripheral arterial tone signals and pulse rate (two important outputs of the autonomic nervous system). The WatchPAT system allows us to determine pulse rate, oxygen saturation, actigraphy data, and the 4% oxygen desaturation index and the respiratory event index, rapid eye movement (REM), light sleep, and deep sleep stages in the form of AHI, REM%, light sleep%, and deep sleep%, respectively (Nittman et al., 2004; Kapur et al., 2017). Furthermore, the WatchPAT system also automatically calculates percentage of time spent in the prone, supine, left side-lying, and right side-lying sleeping positions; the AHI for each sleep depth (i.e., the REM-AHI and NREM-AHI) as well as for each body position (prone, supine, left side-lying, and right side-lying); and number of awakenings during sleep.

### 2.4 Statistics and reproducibility

First, the basic statistics regarding all parameters analysed in this study were performed including all participants and high- and low-BMI groups divided by the median BMI of all participants. A pooled *t*-test was performed to compare age and all sleep-related parameters obtained by the WatchPAT system in two groups: high-BMI and low-BMI; *P*-values were calculated. Second, a linear regression model was created to evaluate whether BMI could be used to predict each of all AHI parameters as continuous variables. Using the linear regression analysis, non-standardized estimation values *β*), *P*-values, and *R* ^2^ were calculated to estimate the effect of each explanatory variable. Third, in each of the high/low-BMI groups and high/low-BMI groups with AHI ≥15/h, we performed multivariate correlation analysis of age and all sleep-related parameters considered in this study; we calculated the Spearman correlation coefficient ρ) and *P*-value. Finally, the parameters that showed interesting relationships using the multivariate correlation analysis were extracted and reevaluated by linear regression analysis. *P*-values < 0.05 were considered statistically significant. These statistical analyses were performed using JMP^®^ Pro 17.0 (SAS Institute Inc., Cary, NC) software.

## 3 Results

### 3.1 Participants

In total, 32 (30.8%) of 104 participants met the YHAB study criteria, none of whom had been diagnosed with SAS or received continuous positive airway pressure treatment prior to the YHAB study. Of the 32 participants, 56% were male. Table 1 shows the participant characteristics, including the sleep and AHI data used in the analysis. The mean values for age and BMI were 86.7 years and 23.5 kg/m^2^, respectively. The mean percentage of rapid eye movement (REM%), light sleep (light sleep%), and deep sleep (deep sleep%) were 21.2%, 64.8%, and 14.0%, respectively. The AHI during REM sleep (REM-AHI; 28.9/h) value was higher than that during non-REM sleep (NREM-AHI; 22.1/h), and the mean AHI value in the supine position (29.6/h) was highest among all four positions.

**TABLE 1:** Characteristics of the participants in the YHAB dataset (N = 32).

Parameter	Mean	SD	SE	Upper 95% mean	Lower 95% mean	N	N Missing
Age (years)	86.7	3.2	0.6	87.8	85.5	32	0
BMI (kg/m^2^)	23.5	3.1	0.6	24.6	22.3	32	0
REM %	21.2	6.6	1.2	23.7	18.8	31	1
Light sleep %	64.8	9.8	1.8	68.4	61.2	31	1
Deep sleep %	14.0	6.5	1.2	16.3	11.6	31	1
AHI (events/h)	23.2	15.4	2.7	28.8	17.7	32	0
REM-AHI (events/h)	28.9	15.6	2.8	34.7	23.1	30	2
NREM-AHI (events/h)	22.1	16.1	2.9	28.1	16.0	30	2
AHI in prone (events/h)	18.4	24.1	12.0	56.7	−20.0	4	28
AHI in supine (events/h)	29.6	18.9	3.4	36.4	22.7	32	0
AHI in left side lying (events/h)	21.2	15.9	3.4	28.3	14.2	22	10
AHI in right side lying (events/h)	17.0	17.8	3.3	23.8	10.2	29	3
Number of awakenings	8.6	5.8	1.0	10.7	6.5	32	0

AHI, apnoea-hypopnea index; BMI, body mass index; N, number of participants for whom results were available in the inspection report; NREM, non-rapid eye movement; REM, rapid eye movement; SD, standard deviation; SE, standard error of the mean; YHAB, Yamanashi Healthy active long-living older people Biobank for healthy ageing biosciences.

### 3.2 Estimation of AHI characteristics using a linear regression model with BMI as the dependent variable

Table 2 shows the AHI characteristics in terms of sleep stage and body position estimated using a linear regression model with BMI as the dependent variable. All estimated values *β*) were positive. The *P* values of the REM-AHI (0.017) and AHI in right side lying (0.046) were significant at <0.05 and N > 10.

**TABLE 2:** Estimated effect of BMI on AHI parameters using the linear regression model.

AHI (events/h)	N	*R* ^2^	Estimate (*β*)	*P*-value
AHI	32	0.11	1.61	0.070
REM-AHI	30	0.19	2.30	**0.017**
NREM-AHI	30	0.12	1.89	0.063
AHI in prone	4	0.90	5.53	0.050
AHI in supine	32	0.13	2.16	**0.046**
AHI in left side lying	22	0.03	0.86	0.431
AHI in right side lying	29	0.15	2.22	**0.042**

AHI, apnoea-hypopnea index; BMI, body mass index; N, number of participants for whom results were available in the inspection report; NREM, non-rapid eye movement; REM, rapid eye movement. Bold font indicates *P*-values <0.05.

### 3.3 Basic comparison between high- and low-BMI groups

**FIGURE 1:**
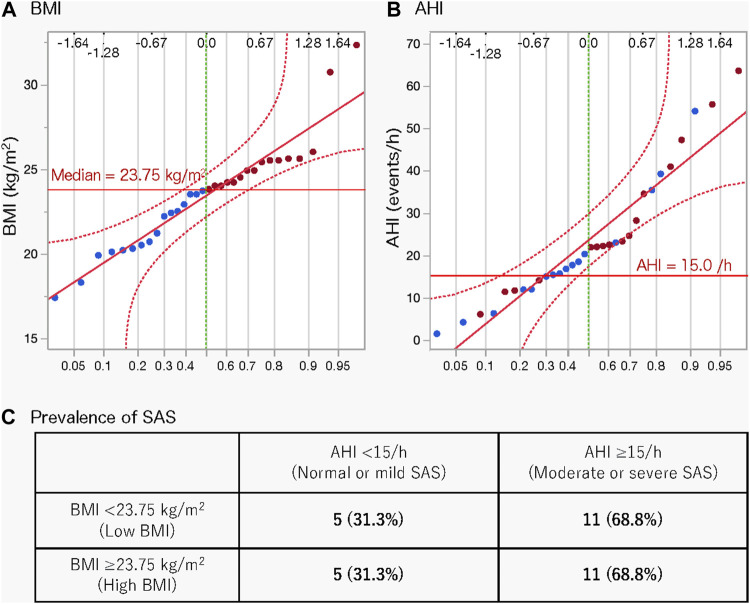
Normal quantile plots of BMI **(A)** and AHI **(B)** and prevalence of SAS according to BMI **(C)**. Red circles indicate a BMI greater than the median value; blue circles indicate a BMI less than the median value. AHI, apnoea-hypopnea index; BMI, body mass index; SAS, sleep apnoea syndrome.

**FIGURE 2:**
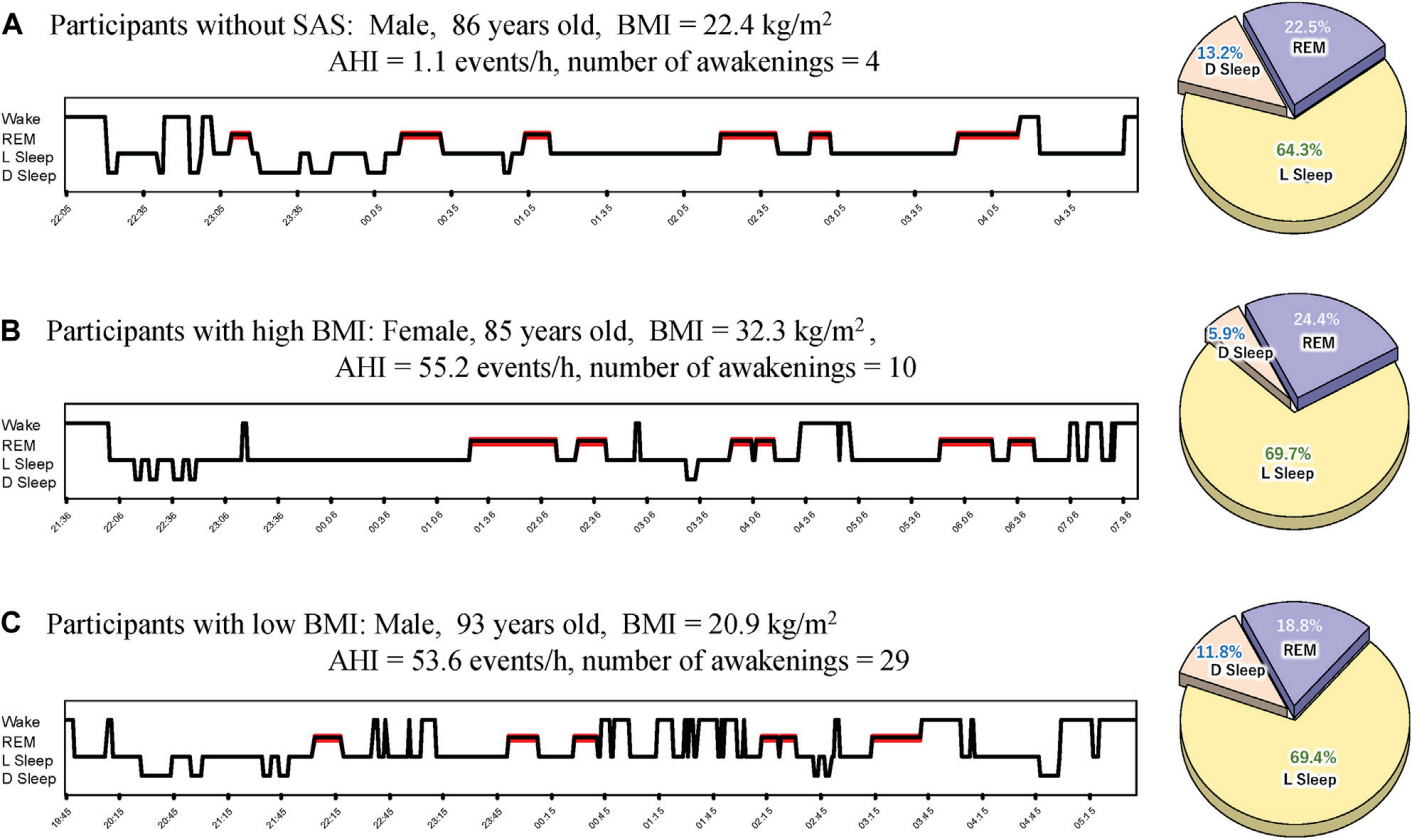
Representative sleep profiles. **(A)** Profile of a participant without SAS (with the lowest AHI), **(B)** a participant with SAS in the high-BMI group, and **(C)** a participant with SAS in the low-BMI group. AHI, apnoea-hypopnea index; BMI, body mass index; D Sleep, deep sleep; L Sleep, light sleep; REM, rapid eye movement; SAS, sleep apnoea syndrome.

We calculated and compared the basic statistics for each parameter between the high- and low-BMI groups divided by the median BMI (23.75 kg/m^2^) (Figure 1A). The male ratio of both groups was the same (0.563). Eleven (68.8%) participants each in the high- and low-BMI groups had moderate or severe sleep apnoea (Figures 1B, C). Figure 2 shows the sleep stages of three representative participants: one without SAS (Figure 2A), one with a high BMI (Figure 2A), and one with a low BMI (Figure 2C). Focusing on the differences in sleep status (percentages of sleep stages, number of awakenings, and AHI) between the high- and low-BMI groups shown in Figure 2, the statistical differences in sleep parameters between groups were examined in detail. Among all the parameters investigated, only the REM-AHI showed a significant difference between the high- and low-BMI groups, both among all participants (35.1/h vs. 21.9/h, respectively, *p* = 0.005; Table 3) and among those with an AHI ≥15/h (40.8/h vs. 27.7/h, respectively, *p* = 0.018; Table 4). The male ratios of the compared parameters in the low- and high-BMI groups were 0.38–0.5 and 0.58–0.79, respectively, for all participants (Table 3), and 0.33–0.44 and 0.56–0.80, respectively, for participants with an AHI ≥15/h (Table 4).

**TABLE 3:** Comparisons between all participants in the high- and low-BMI groups.

Parameter	BMI	N	Male ratio	Mean	SD	Lower 95% mean	Upper 95% mean	Difference (high BMI–low BMI)	*P*-value
Age (years)	low	16	0.44	86.7	3.4	84.9	88.5	−0.1	0.970
	high	16	0.69	86.6	3.1	84.7	88.3		
REM %	low	15	0.47	20.3	7.2	16.4	24.3	1.8	0.465
	high	16	0.69	22.1	6.1	18.8	25.3		
Light sleep %	low	15	0.47	67.9	9.8	62.5	73.4	−6.0	0.138
	high	16	0.69	61.9	9.2	57.0	66.8		
Deep sleep %	low	15	0.47	11.7	5.3	8.8	14.7	4.3	0.086
	high	16	0.69	16.0	7.0	12.3	19.8		
AHI (events/h)	low	16	0.44	18.8	13.5	11.6	26.0	8.9	0.080
	high	16	0.69	27.7	16.3	19.0	36.4		
REM-AHI (events/h)	low	14	0.50	21.9	11.5	15.2	28.5	13.2	**0.005**
	high	16	0.69	35.1	16.3	26.5	43.8		
NREM-AHI (events/h)	low	14	0.50	17.6	14.1	9.5	25.7	8.4	0.119
	high	16	0.69	26.0	17.2	16.8	35.1		
AHI in supine (events/h)	low	16	0.44	25.9	19.7	15.4	36.3	7.4	0.193
	high	16	0.69	33.3	18.0	23.6	42.9		
AHI in left side lying (events/h)	low	10	0.50	18.8	14.5	8.4	29.1	4.5	0.575
	high	12	0.58	23.3	17.4	12.2	34.3		
AHI in right side lying (events/h)	low	15	0.38	11.7	15.0	3.4	19.9	11.0	0.058
	high	14	0.79	22.7	19.4	11.5	33.9		
Number of awakenings	low	16	0.44	9.4	7.2	5.5	13.2	−1.6	0.806
	high	16	0.69	7.8	4.0	5.7	10.0		

The pooled *t*-test was used. AHI, in the prone position was not included because the number of participants was small. Low BMI: <23.75 kg/m^2^; high BMI: ≥23.75 kg/m^2^. Bold font indicates *P*-values <0.05.

AHI, apnoea-hypopnea index; BMI, body mass index; h, hour; N, number of participants for whom results were available in the inspection report; NREM, non-rapid eye movement; REM, rapid eye movement; SD, standard deviation.

**TABLE 4:** Comparisons between the participants in the high- and low-BMI groups with an AHI ≥15/h in the YHAB dataset.

Parameter	BMI	N	Male ratio	Mean	SD	Lower 95% mean	Upper 95% mean	Difference (high BMI–low BMI)	*P*-value
BMI	low	10	0.40	21.4	2.2	19.7	23.1	4.1	**0.001**
	high	12	0.73	25.8	2.7	24.3	27.3		
Age (years)	low	10	0.40	87.3	4.0	85.1	89.5	−0.9	0.396
	high	12	0.73	86.1	2.5	84.1	88.1		
REM%	low	9	0.44	20.2	6.8	16.0	24.4	1.0	0.313
	high	12	0.73	23.0	5.3	19.3	26.6		
Light sleep %	low	9	0.44	69.1	10.8	62.7	75.4	−1.9	0.071
	high	12	0.73	61.3	7.8	55.8	66.9		
Deep sleep %	low	9	0.44	10.7	6.3	5.9	15.6	1.6	0.122
	high	12	0.73	15.7	7.4	11.5	19.9		
AHI (events/h)	low	10	0.40	25.2	13.0	16.0	34.4	1.4	0.181
	high	12	0.73	33.5	14.6	25.1	41.8		
REM-AHI (events/h)	low	9	0.44	27.7	9.1	19.6	35.7	2.6	**0.018**
	high	12	0.73	40.8	12.9	33.8	47.7		
NREM-AHI (events/h)	low	9	0.44	22.8	14.6	11.9	33.7	1.3	0.224
	high	12	0.73	31.5	16.3	22.0	41.0		
AHI in supine (events/h)	low	10	0.40	35.1	3.0	24.1	46.1	0.7	0.475
	high	12	0.73	40.3	36.1	30.2	50.3		
AHI in left side lying (events/h)	low	7	0.43	21.6	18.5	7.5	35.6	0.7	0.489
	high	9	0.56	27.8	15.0	15.4	40.2		
AHI in right side lying (events/h)	low	9	0.33	17.6	16.5	4.5	30.6	1.1	0.276
	high	11	0.80	27.0	18.0	15.2	38.7		
Number of awakenings	low	10	0.40	10.7	17.1	6.5	14.9	−1.0	0.348
	high	12	0.73	8.1	19.7	4.3	11.9		

The pooled *t*-test was used. AHI, in the prone position was not included because the number of participants was small. Low BMI: <23.75 kg/m^2^; high BMI: ≥23.75 kg/m^2^. Bold font indicates *P*-values <0.05.

AHI, apnoea-hypopnea index; BMI, body mass index; h, hour; N, number of participants for whom results were available in the inspection report; NREM, non-rapid eye movement; REM, rapid eye movement; SD, standard deviation; YHAB, Yamanashi Healthy active long-living older people Biobank for healthy ageing biosciences.

### 3.4 Effects of age and sleep parameters at different sleep stages in the high- and low-BMI groups

**FIGURE 3:**
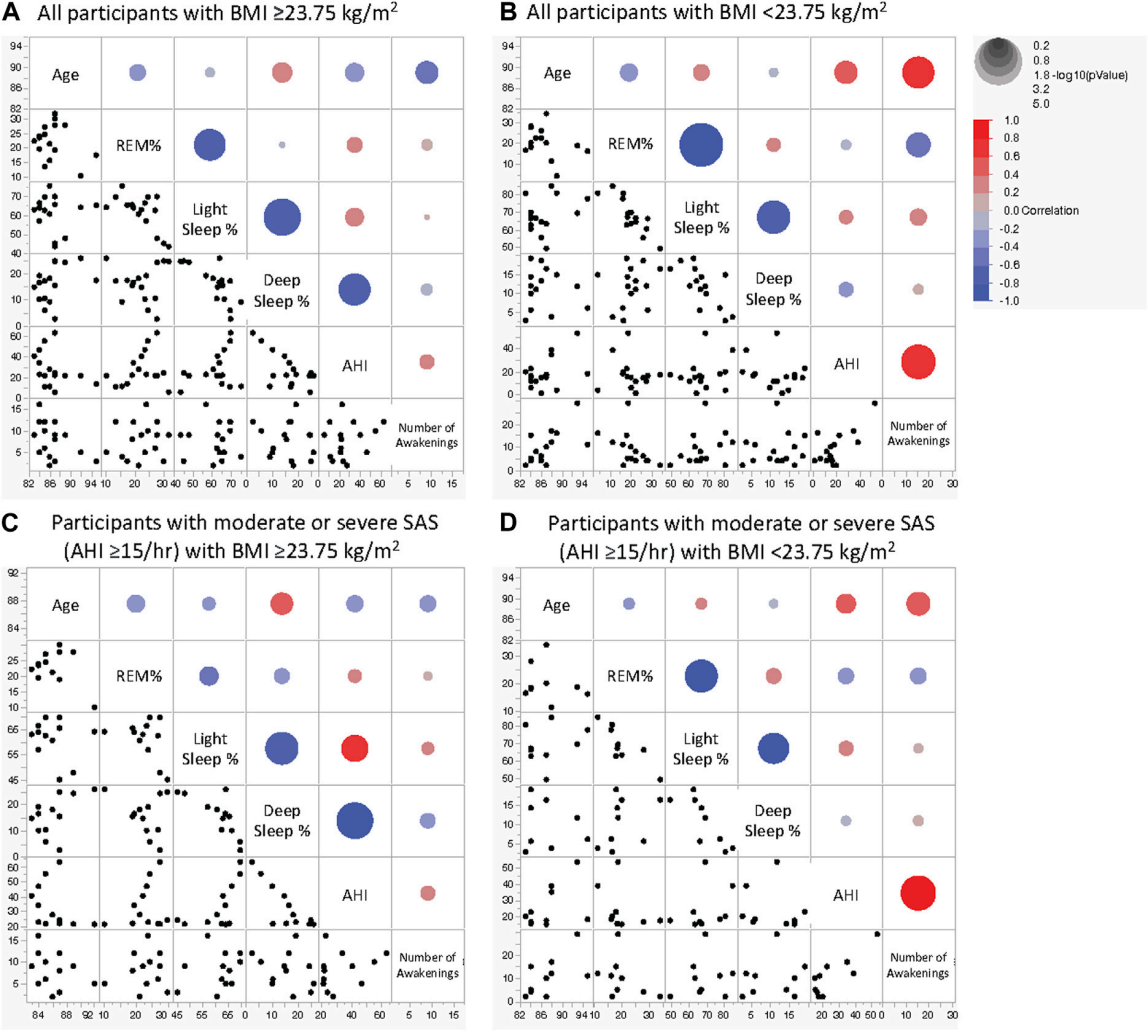
Scatterplot matrix for indicators of age and sleep parameters in older adults. Sleep stage percentage, AHI, and the number of awakenings in all participants with BMI ≥23.75 kg/m^2^ **(A)**, all participants with BMI <23.75 kg/m^2^ **(B)**, participants with moderate or severe SAS with BMI ≥23.75 kg/m^2^ **(C)**, and participants with moderate or severe SAS with BMI <23.75 kg/m^2^ **(D)**. The colour of the circles in the scatterplots indicates the correlation coefficient, and their size indicates the non-parametric density. Blue and red represent negative and positive correlation coefficients, respectively. The larger the circle, the smaller the *P*-value. AHI, apnoea-hypopnea index; BMI, body mass index; REM, rapid eye movement; NREM, non-rapid eye movement; SAS, sleep apnoea syndrome.

Multivariate correlation analysis (Figure 3; Table 5) findings differed between all the participants in the high-BMI group (Figure 3A), all the participants in the low-BMI group (Figure 3B), participants with an AHI ≥15/h with a high BMI (Figure 3C), and participants with an AHI ≥15/h with a low BMI (Figure 3D) as follows. All participants and participants with an AHI ≥15/h in the high-BMI group showed a significant negative correlation between deep sleep% and AHI (ρ = 0.503 and *p* = 0.047 for all participants and ρ = −0.748 and *p* = 0.005 for patients with an AHI ≥15/h); however, this was not observed in participants in the low-BMI group (ρ = −0.218 and *p* = 0.435 for all participants and ρ = −0.250 and *p* = 0.517 for patients with an AHI ≥15/h). All participants in the low-BMI group demonstrated a significant positive correlation between the number of awakenings and age (ρ = 0.651 and *p* = 0.006); however, this was not observed in participants in the low-BMI group with an AHI ≥15/h (ρ = 0.616 and *p* = 0.058) or participants in the high-BMI group (ρ = −0.420 and *p* = 0.105 for all participants and ρ = −0.332 and *p* = 0.292 for patients with an AHI ≥15/h). Participants in the low-BMI group with an AHI ≥15/h showed a significant positive correlation between the number of awakenings and AHI (ρ = 0.675 and *p* = 0.032); however, this was not observed in all participants in the low-BMI group (ρ = 0.374 and *p* = 0.155) or those in the high-BMI group (ρ = 0.188 and *p* = 0.485 for all participants and ρ = 0.288 and *p* = 0.364 for patients with an AHI ≥15/h). Although all participants in the low-BMI group presented a significant negative correlation between the number of awakenings and REM% (ρ = −0.628 and *p* = 0.012), this was not observed in participants in the low-BMI group with an AHI ≥15/h (ρ = −0.385 and *p* = 0.306) or participants in the high-BMI group (ρ = 0.164 and *p* = 0.543 for all participants and ρ = 0.205 and *p* = 0.744 for patients with an AHI ≥15/h). All participants in the low-BMI group demonstrated a significant negative correlation between light sleep% and REM% (ρ = −0.796 and *p* < 0.001 for all participants and ρ = −0.783 and *p* = 0.013 for those with an AHI ≥15/h); however, patients in the high-BMI group showed a smaller negative correlation between light sleep% and REM% than that shown by those in the low-BMI group, with statistical significance for all participants (ρ = −0.535 and *p* = 0.033), and without statistical significance for those with an AHI ≥15/h (ρ = −0.322 and *p* = 0.308). A property common to all groups was the predominant negative correlation between deep sleep% and light sleep%.

**TABLE 5:** Correlation coefficients of indicators of age and sleep parameters in older adults.

Variable	By variable	High BMI (≥23.75 kg/m^2^)				Low BMI (<23.75 kg/m^2^)			
		All		Moderate or severe SAS (AHI >15/h)		All		Moderate or severe SAS (AHI >15/h)	
		Spearman ρ	*P*-value	Spearman ρ	*P*-value	Spearman ρ	*P*-value	Spearman ρ	*P*-value
REM %	Age	0.002	0.996	0.117	0.717	−0.295	0.286	−0.170	0.661
AHI	Age	−0.446	0.083	−0.505	0.094	0.241	0.369	0.426	0.220
Number of awakenings	Age	−0.420	0.105	−0.332	0.292	**0.651**	**0.006**	0.616	0.058
Light sleep %	Age	−0.090	0.742	−0.064	0.844	0.211	0.450	0.204	0.598
Deep sleep %	Age	0.431	0.096	0.426	0.167	0.109	0.698	0.043	0.913
Number of awakenings	AHI	0.188	0.485	0.288	0.364	0.374	0.154	**0.675**	**0.032**
AHI	Deep sleep %	**−0.503**	**0.047**	**−0.748**	**0.005**	−0.218	0.435	−0.250	0.517
Number of awakenings	Deep sleep %	−0.212	0.431	−0.323	0.305	0.167	0.552	0.193	0.620
AHI	Light sleep %	0.200	0.458	0.559	0.059	0.164	0.559	0.450	0.224
Number of awakenings	Light sleep %	0.102	0.706	0.250	0.434	0.370	0.175	0.176	0.651
Deep sleep %	Light sleep %	**−0.756**	**0.001**	**−0.727**	**0.007**	**−0.686**	**0.005**	**−0.867**	**0.003**
AHI	REM %	0.277	0.300	0.161	0.618	−0.300	0.277	−0.500	0.171
Number of awakenings	REM %	0.164	0.543	0.105	0.744	**−0.628**	**0.012**	−0.385	0.306
Light sleep %	REM %	**−0.535**	**0.033**	−0.322	0.308	**−0.796**	**0.000**	**−0.783**	**0.013**
Deep sleep %	REM %	0.050	0.854	−0.140	0.665	0.229	0.413	0.483	0.188

Blue cells show negative correlation coefficients, and red cells show positive correlation coefficients with P-value <0.05. Bold font indicates *P*-values <0.05, and bold with underlined font indicates *P*-values < 0.01. AHI, apnoea-hypopnea index; BMI, body mass index; REM, rapid eye movement; SAS, sleep apnoea syndrome.

### 3.5 Estimation of age, deep sleep%, and number of awakenings using BMI

The high- and low-BMI groups were reanalysed using a linear regression model with respect to age (Table 6), deep sleep% (Table 7), and number of awakenings (Table 8), as the multivariate correlation analysis (Figure 3; Table 5) suggested that the relationships of these variables with AHI may differ between groups. As shown in the age estimation results (Table 6), *β* was >0 in the REM-AHI and AHI in the right side-lying position in the low-BMI group and showed statistical significance (*p* < 0.05; Table 6). Regarding deep sleep% analysis (Table 7), *β* was <0 in the AHI, REM-AHI, NREM-AHI, and AHI in the supine and right side-lying positions, with statistical significance (*p* < 0.05) in the high-BMI group. In the analysis of the number of awakenings (Table 8), *β* was >0 in all variables in the low-BMI group, with statistical significance (*p* < 0.05).

**TABLE 6:** Estimated effect of age on AHI characteristics in the high- and low-BMI groups using the linear regression model.

BMI	Parameter (events/h)	N	*R* ^2^	Estimate (*β*)	*P*-value
Low BMI (<23.75 kg/m^2^)	AHI	16	0.20	0.11	0.081
	REM-AHI	14	0.32	0.18	**0.033**
	NREM-AHI	14	0.26	0.13	0.062
	AHI in supine	16	0.14	0.06	0.155
	AHI in left side lying	10	0.15	0.11	0.264
	AHI in right side lying	15	0.41	0.15	**0.010**
High BMI (≥23.75 kg/m^2^)	AHI	16	0.12	−0.07	0.196
	REM-AHI	16	0.03	−0.04	0.498
	NREM-AHI	16	0.12	−0.06	0.189
	AHI in supine	16	0.05	−0.04	0.394
	AHI in left side lying	12	0.08	−0.05	0.383
	AHI in right side lying	14	0.20	−0.05	0.111

AHI, apnoea-hypopnea index; BMI, body mass index; REM, rapid eye movement; N, number of participants for whom results were available in the inspection report; NREM, non-rapid eye movement. Bold font indicates *P*-values < 0.05, and bold with underlined font indicates *P*-values < 0.01.

**TABLE 7:** Estimated effect of deep sleep% on AHI characteristics in the high- and low-BMI groups using the linear regression model.

BMI	Parameter (events/h)	N	*R* ^2^	Estimate (*β*)	*P*-value
Low BMI (<23.75 kg/m^2^)	AHI	15	0.06	−0.09	0.399
	REM-AHI	14	0.03	−0.09	0.530
	NREM-AHI	14	0.03	−0.07	0.526
	AHI in supine	15	0.11	−0.09	0.217
	AHI in left side lying	10	0.01	0.04	0.771
	AHI in right side lying	14	0.01	0.03	0.764
High BMI (≥23.75 kg/m^2^)	AHI	16	0.42	−0.28	**0.006**
	REM-AHI	16	0.27	−0.22	**0.040**
	NREM-AHI	16	0.44	−0.27	**0.005**
	AHI in supine	16	0.27	−0.20	**0.038**
	AHI in left side lying	12	0.22	−0.16	0.122
	AHI in right side lying	14	0.56	−0.29	**0.002**

AHI, apnoea-hypopnea index; BMI, body mass index; REM, rapid eye movement; N, number of participants for whom results were available in the inspection report; NREM, non-rapid eye movement. Bold font indicates *P*-values < 0.05, and bold with underlined font indicates *P*-values < 0.01.

**TABLE 8:** Estimated effect of the number of awakenings during sleep on AHI characteristics in the high- and low-BMI groups using the linear regression model.

BMI	Parameter (events/h)	N	*R* ^2^	Estimate (*β*)	*P*-value
Low BMI (<23.75 kg/m^2^)	AHI	16	0.49	0.37	**0.003**
	REM-AHI	14	0.54	0.45	**0.003**
	NREM-AHI	14	0.67	0.41	**0.000**
	AHI in supine	16	0.33	0.21	**0.019**
	AHI in left side lying	10	0.57	0.41	**0.012**
	AHI in right side lying	15	0.36	0.29	**0.017**
High BMI (≥23.75 kg/m^2^)	AHI	16	0.05	0.06	0.389
	REM-AHI	16	0.00	0.00	0.943
	NREM-AHI	16	0.08	0.07	0.277
	AHI in supine	16	0.02	0.03	0.623
	AHI in left side lying	12	0.02	0.04	0.651
	AHI in right side lying	14	0.05	0.05	0.440

AHI, apnoea-hypopnea index; BMI, body mass index; REM, rapid eye movement; N, number of participants for whom results were available in the inspection report; NREM, non-rapid eye movement. Bold font indicates *P*-values < 0.05, and bold with underlined font indicates *P*-values < 0.01.

## 4 Discussion

This study examined the relationships among BMI, sleep parameters (including AHI, sleep stages, and number of awakenings), and age in older adults who were in relatively good health and had no previous diagnosis of SAS. The prevalence of moderate or severe SAS was the same in the low- and high-BMI groups (both 68.8%). In all examined sleep parameters, AHI tended to increase with increasing BMI, with a significant increase during REM sleep. This study found the following differences between the high- and low-BMI groups: 1) in the high-BMI group, deep sleep% tended to decrease as AHI increased, with no significant correlation between the number of awakenings and AHI and 2) in the low-BMI group, there was a tendency for the number of awakenings to increase with age and AHI, although no significant correlation existed between deep sleep% and AHI.

We found that the prevalence of SAS (68.8%) in the low-BMI group was as high as that in the high-BMI group. This result is generally consistent with a small long-term longitudinal study conducted in San Francisco (Bliwise et al., 2010) and a large cross-sectional study conducted in Nagahama (Matsumoto et al., 2020). We also examined the sleep conditions in detail to determine the influence of BMI on SAS. Overall, the AHI tended to increase with increasing BMI in all states of sleep, with REM sleep and sleeping in the supine position showing the highest correlation with BMI; these findings are consistent with those of other studies (Oksenberg et al., 2010; Koo et al., 2018; Park et al., 2022). In addition, the REM-AHI was found to be the most strongly positively correlated with BMI, which was similar to findings reported in other studies (Acosta-Castro et al., 2018; Falla et al., 2023). In contrast, the current study showed that patients with SAS who have high BMI tend to have shallow sleep without awakenings and that the sleep of patients with SAS who have low BMI tends to be fragmented with awakenings, although deep sleep is achieved. Our findings regarding differences in sleep status between older adults with high and low BMI may significantly contribute to the literature, as we found no previous studies reporting these findings. However, our sample was as small as that of the San Francisco study; the fact that several of our findings are consistent with those of similar previous studies supports the validity of our findings regarding differences in sleep status.

Generally, increases in AHI have been attributed to airway narrowing due to excessive fat deposition (Peppard et al., 2013; White and Bradley, 2013; Pépin et al., 2016); however, this explanation is not applicable to patients with low BMI who have SAS. Other factors, such as tongue-based airway obstruction (Vanderveken et al., 2013; Strollo et al., 2014) and central nerve abnormalities (Oldenburg et al., 2007; Oldenburg et al., 2009; Cowie et al., 2015), are associated with SAS. Bliwise et al. (2010) noted in their discussion that the increase in AHI with ageing and weight loss could be due to muscle mass loss, but no muscle mass measurements or muscle-related motor function were reported. Our previous study, which included the same 32 participants as those in this study, showed that decline in motor function correlated with increased AHI (Tanaka et al., 2022) and worsening of SAS severity correlated with age in the low-BMI group. Loss of weight, muscle mass, and motor function with ageing is part of the typical pathophysiology of frailty (Bliwise et al., 2010; Clegg et al., 2013). The prevalence of SAS is higher in participants who are frail than in those who are not (Ensrud et al., 2009; Ensrud et al., 2012; Piovezan et al., 2013). Patients who are frail have also been reported to have an increased number and duration of awakenings than that observed in healthy older adults, consistent with the increased number of awakenings observed in the low-BMI group in this study (Ensrud et al., 2009). It has been well-documented that the number of awakenings during sleep increases with age (Knowles and MacLean, 1990; Edwards et al., 2010). Similarly, this study included participants older than 80 years, and only a few of them reported having a good sleep status, indicated by the number of awakenings and sleep depth. These findings suggest that patients with SAS who have low BMI analysed in this study may be frail due to physical ageing, including the loss of strength in respiratory muscles.

In our study, when restricted to older adults with high BMI, AHI did not increase with ageing but rather decreased, although this finding was not statistically significant. This is in direct opposition to the finding for patients with SAS with low BMI, and to the best of our knowledge, this has not been reported by any study focused on the difference in sleep status according to BMI. The older adults with high BMI in our study might have been those with good health and maintained muscle mass. The SAS patients with high BMI in this study might have been older adults who have successfully maintained their health by preventing physical ageing, including loss of respiratory muscle strength.

Another important study finding was the lack of awareness of SAS among the study participants. SAS has been shown to increase not only mortality but also drowsiness, which can lead to decreased alertness and traffic accidents (Findley et al., 1989; Philip et al., 2008). Therefore, increasing the awareness of SAS is essential for early detection and treatment (Aurora et al., 2012; Martínez-García et al., 2012; Jennum et al., 2015; Dodds et al., 2020). Our study highlights the importance of considering SAS equally in older adults with low BMI as well as in those with high BMI. Since patients with SAS with low BMI are particularly likely to have associated frailty, it is necessary to consider snoring and other symptoms associated with SAS as indicators of frailty.

This study had some limitations. First, the study population was limited to the dataset obtained from the Yamanashi Healthy active long-living older people Biobank for healthy ageing biosciences survey, which was smaller than that in other SAS studies (Young et al., 2002; Jordan et al., 2014; Acosta-Castro et al., 2018; Matsumoto et al., 2020). However, we found that the basic characteristics of the study population, such as sleep stage and the AHI for each body position, were similar to those reported in larger studies, which partially confirms the validity of our results. Nonetheless, similar analyses with larger cohorts are required to further confirm the generalisability of our findings. Second, the participants in this study were limited to those from Yamanashi Prefecture; thus, it is necessary to verify whether the same results can be observed in other regions or countries. Third, standardising the use of common clothing throughout the year when measuring weights was impossible. This was because the study was conducted during the time of COVID-19 infection outbreak and ventilation and other measures were taken, particularly during the winter months. Therefore, asking older adults to change into uniform clothing was impossible. Although we asked them to undress as lightly as possible, a slight concern remains regarding the accuracy of BMI compared with physical examination. Lastly, WatchPAT, which was used in this study, is not equipped with a function to perform PSG; therefore, not only can it not detect central nervous system abnormalities that are thought to be associated with SAS but also the evaluation of REM and NREM sleep is an estimated evaluation not based on PSG. The use of PSG was avoided in this study in consideration of the burden on the older adults. We hope that the exploratory findings obtained with the less burdensome WatchPAT used in this study will be followed up with detailed validation studies using PSG and other instruments.

## Data Availability

The original contributions presented in the study are included in the article/Supplementary Material, further inquiries can be directed to the corresponding author.

## Supplementary material

SUPPLEMENTARY FIGURE S1 Normal quantile plots of BMI **(A)** and AHI **(B)**, and prevalence of SAS according to BMI **(C)**.

SUPPLEMENTARY TABLE S1 Estimated effect of BMI on AHI parameters using the linear regression model.

SUPPLEMENTARY TABLE S2 Comparisons between all participants in the high- and low-BMI groups.

SUPPLEMENTARY TABLE S3 Comparisons between the participants in the high- and low-BMI groups with an AHI ≥15/h in the YHAB dataset.

SUPPLEMENTARY TABLE S4 Correlation coefficients of indicators of age and sleep parameters in older adults.

## Abbreviations

AHI, apnoea-hypopnoea index; BMI, body mass index; SAS, sleep apnoea syndrome; PSG, polysomnography; REM, rapid eye movement.
