# Supplementary material for: Sleep status of older adults with sleep apnoea syndrome may vary by body mass index
Source: Front Aging. 2024 May 1;5:1331448. doi: 10.3389/fragi.2024.1331448 (PMC11094249; doi:10.3389/fragi.2024.1331448)
Supplement: Supplementary file 1 [file DataSheet1.docx]

Supplementary Material

.

# Supplementary Figures

**Supplementary Figure 1.** Normal quantile plots of BMI (A) and AHI (B), and prevalence of SAS according to BMI (C). Red circles indicate a BMI greater than the median value; blue circles indicate a BMI less than the median value. AHI, apnoea-hypopnea index; BMI, body mass index; SAS, sleep apnoea syndrome.

# Supplementary Tables

**Supplementary Table 1.** Estimated effect of BMI on AHI parameters using the linear regression model

| **AHI (events/h)** | **N** | ***R*^2^** | **Estimate (***β***)** | ***P*-value** |
| --- | --- | --- | --- | --- |
| AHI | 32 | 0.11 | 1.61 | 0.070 |
| REM-AHI | 30 | 0.19 | 2.30 | **0.017** |
| NREM-AHI | 30 | 0.12 | 1.89 | 0.063 |
| AHI in prone | 4 | 0.90 | 5.53 | **0.050** |
| AHI in supine | 32 | 0.13 | 2.16 | **0.046** |
| AHI in left side lying | 22 | 0.03 | 0.86 | 0.431 |
| AHI in right side lying | 29 | 0.15 | 2.22 | **0.042** |

Bold font indicates *P*-values < 0.05.

AHI, apnoea-hypopnea index; BMI, body mass index; N, number; NREM, non-rapid eye movement; REM, rapid eye movement.

**Supplementary Table 2.** Comparisons between all participants in the high- and low-BMI groups

| **Parameter** | **BMI** | **N** | **Male**  **Ratio** | **Mean** | **SD** | **Lower 95% Mean** | **Upper 95%**  **Mean** | **Difference**  **(high BMI – low BMI)** | ***P*-value** |
| --- | --- | --- | --- | --- | --- | --- | --- | --- | --- |
| Age (years) | low | 16 | 0.44 | 86.7 | 3.4 | 84.9 | 88.5 | -0.1 | 0.970 |
|  | high | 16 | 0.69 | 86.6 | 3.1 | 84.7 | 88.3 |  |  |
| REM % | low | 15 | 0.47 | 20.3 | 7.2 | 16.4 | 24.3 | 1.8 | 0.465 |
|  | high | 16 | 0.69 | 22.1 | 6.1 | 18.8 | 25.3 |  |  |
| Light sleep % | low | 15 | 0.47 | 67.9 | 9.8 | 62.5 | 73.4 | -6.0 | 0.138 |
|  | high | 16 | 0.69 | 61.9 | 9.2 | 57.0 | 66.8 |  |  |
| Deep sleep % | low | 15 | 0.47 | 11.7 | 5.3 | 8.8 | 14.7 | 4.3 | 0.086 |
|  | high | 16 | 0.69 | 16.0 | 7.0 | 12.3 | 19.8 |  |  |
| AHI (events/h) | low | 16 | 0.44 | 18.8 | 13.5 | 11.6 | 26.0 | 8.9 | 0.080 |
|  | high | 16 | 0.69 | 27.7 | 16.3 | 19.0 | 36.4 |  |  |
| REM-AHI (events/h) | low | 14 | 0.50 | 21.9 | 11.5 | 15.2 | 28.5 | 13.2 | **0.005** |
|  | high | 16 | 0.69 | 35.1 | 16.3 | 26.5 | 43.8 |  |  |
| NREM-AHI (events/h) | low | 14 | 0.50 | 17.6 | 14.1 | 9.5 | 25.7 | 8.4 | 0.119 |
|  | high | 16 | 0.69 | 26.0 | 17.2 | 16.8 | 35.1 |  |  |
| AHI in supine (events/h) | low | 16 | 0.44 | 25.9 | 19.7 | 15.4 | 36.3 | 7.4 | 0.193 |
|  | high | 16 | 0.69 | 33.3 | 18.0 | 23.6 | 42.9 |  |  |
| AHI in left side lying (events/h) | low | 10 | 0.50 | 18.8 | 14.5 | 8.4 | 29.1 | 4.5 | 0.575 |
|  | high | 12 | 0.58 | 23.3 | 17.4 | 12.2 | 34.3 |  |  |
| AHI in right side lying (events/h) | low | 15 | 0.38 | 11.7 | 15.0 | 3.4 | 19.9 | 11.0 | 0.058 |
|  | high | 14 | 0.79 | 22.7 | 19.4 | 11.5 | 33.9 |  |  |
| Number of awakenings | low | 16 | 0.44 | 9.4 | 7.2 | 5.5 | 13.2 | -1.6 | 0.806 |
|  | high | 16 | 0.69 | 7.8 | 4.0 | 5.7 | 10.0 |  |  |
|  |  |  |  |  |  |  |  |  |  |

The pooled t-test was used. AHI in the prone position was not included because the number of participants was small. Low BMI: <23.75 kg/m^2^; high BMI: ≥23.75 kg/m^2^. Bold font indicates *P*-values < 0.05.

AHI, apnoea-hypopnea index; BMI, body mass index; h, hour; N, number; NREM, non-rapid eye movement; REM, rapid eye movement; SD, standard deviation.

**Supplementary Table 3.** Comparisons between the participants in the high- and low-BMI groups with an AHI ≥15/h in the YHAB dataset

| Parameter | BMI | N | Male  Ratio | Mean | SD | Lower 95%  Mean | Upper 95%  Mean | Difference  (high BMI  – low BMI) | *P*-value |
| --- | --- | --- | --- | --- | --- | --- | --- | --- | --- |
| BMI | low | 10 | 0.40 | 21.4 | 2.2 | 19.7 | 23.1 | 4.1 | 0.001 |
|  | high | 12 | 0.73 | 25.8 | 2.7 | 24.3 | 27.3 |  |  |
| Age (years) | low | 10 | 0.40 | 87.3 | 4.0 | 85.1 | 89.5 | -0.9 | 0.396 |
|  | high | 12 | 0.73 | 86.1 | 2.5 | 84.1 | 88.1 |  |  |
| REM% | low | 9 | 0.44 | 20.2 | 6.8 | 16.0 | 24.4 | 1.0 | 0.313 |
|  | high | 12 | 0.73 | 23.0 | 5.3 | 19.3 | 26.6 |  |  |
| Light sleep % | low | 9 | 0.44 | 69.1 | 10.8 | 62.7 | 75.4 | -1.9 | 0.071 |
|  | high | 12 | 0.73 | 61.3 | 7.8 | 55.8 | 66.9 |  |  |
| Deep sleep % | low | 9 | 0.44 | 10.7 | 6.3 | 5.9 | 15.6 | 1.6 | 0.122 |
|  | high | 12 | 0.73 | 15.7 | 7.4 | 11.5 | 19.9 |  |  |
| AHI (events/h) | low | 10 | 0.40 | 25.2 | 13.0 | 16.0 | 34.4 | 1.4 | 0.181 |
|  | high | 12 | 0.73 | 33.5 | 14.6 | 25.1 | 41.8 |  |  |
| REM-AHI (events/h) | low | 9 | 0.44 | 27.7 | 9.1 | 19.6 | 35.7 | 2.6 | 0.018 |
|  | high | 12 | 0.73 | 40.8 | 12.9 | 33.8 | 47.7 |  |  |
| NREM-AHI (events/h) | low | 9 | 0.44 | 22.8 | 14.6 | 11.9 | 33.7 | 1.3 | 0.224 |
|  | high | 12 | 0.73 | 31.5 | 16.3 | 22.0 | 41.0 |  |  |
| AHI in supine (events/h) | low | 10 | 0.40 | 35.1 | 3.0 | 24.1 | 46.1 | 0.7 | 0.475 |
|  | high | 12 | 0.73 | 40.3 | 36.1 | 30.2 | 50.3 |  |  |
| AHI in left side lying (events/h) | low | 7 | 0.43 | 21.6 | 18.5 | 7.5 | 35.6 | 0.7 | 0.489 |
|  | high | 9 | 0.56 | 27.8 | 15.0 | 15.4 | 40.2 |  |  |
| AHI in right side lying (events/h) | low | 9 | 0.33 | 17.6 | 16.5 | 4.5 | 30.6 | 1.1 | 0.276 |
|  | high | 11 | 0.80 | 27.0 | 18.0 | 15.2 | 38.7 |  |  |
| Number of awakenings | low | 10 | 0.40 | 10.7 | 17.1 | 6.5 | 14.9 | -1.0 | 0.348 |
|  | high | 12 | 0.73 | 8.1 | 19.7 | 4.3 | 11.9 |  |  |
|  |  |  |  |  |  |  |  |  |  |

The pooled t-test was used. AHI in the prone position was not included because the number of participants was small. Low BMI: <23.75 kg/m^2^; high BMI: ≥23.75 kg/m^2^. Bold font indicates *P*-values < 0.05.

AHI, apnoea-hypopnea index; BMI, body mass index; h, hour; N, number; NREM, non-rapid eye movement; REM, rapid eye movement; SD, standard deviation; YHAB, Yamanashi Healthy active long-living older people Biobank for healthy ageing biosciences.

**Supplementary Table 4.** Correlation coefficients of indicators of age and sleep parameters in older adults

| Variable | By Variable | High BMI (≥23.75 kg/m^2^) | | | | Low BMI (<23.75 kg/m^2^) | | | |
| --- | --- | --- | --- | --- | --- | --- | --- | --- | --- |
|  |  | All | | Moderate or Severe SAS (AHI >15/h) | | All | | Moderate or Severe SAS (AHI >15/h) | |
|  |  | Spearman ρ | *P*-value | Spearman ρ | *P*-value | Spearman ρ | *p*-value | Spearman ρ | *P*-value |
| REM % | Age | 0.002 | 0.996 | 0.117 | 0.717 | -0.295 | 0.286 | -0.170 | 0.661 |
| AHI | Age | -0.446 | 0.083 | -0.505 | 0.094 | 0.241 | 0.369 | 0.426 | 0.220 |
| Number of awakenings | Age | -0.420 | 0.105 | -0.332 | 0.292 | **0.651** | **0.006** | 0.616 | 0.058 |
| Light sleep % | Age | -0.090 | 0.742 | -0.064 | 0.844 | 0.211 | 0.450 | 0.204 | 0.598 |
| Deep sleep % | Age | 0.431 | 0.096 | 0.426 | 0.167 | 0.109 | 0.698 | 0.043 | 0.913 |
| Number of awakenings | AHI | 0.188 | 0.485 | 0.288 | 0.364 | 0.374 | 0.154 | **0.675** | **0.032** |
| AHI | Deep sleep % | **-0.503** | **0.047** | **-0.748** | **0.005** | -0.218 | 0.435 | -0.250 | 0.517 |
| Number of awakenings | Deep sleep % | -0.212 | 0.431 | -0.323 | 0.305 | 0.167 | 0.552 | 0.193 | 0.620 |
| AHI | Light sleep % | 0.200 | 0.458 | 0.559 | 0.059 | 0.164 | 0.559 | 0.450 | 0.224 |
| Number of awakenings | Light sleep % | 0.102 | 0.706 | 0.250 | 0.434 | 0.370 | 0.175 | 0.176 | 0.651 |
| Deep sleep % | Light sleep % | **-0.756** | **0.001** | **-0.727** | **0.007** | **-0.686** | **0.005** | **-0.867** | **0.003** |
| AHI | REM % | 0.277 | 0.300 | 0.161 | 0.618 | -0.300 | 0.277 | -0.500 | 0.171 |
| Number of awakenings | REM % | 0.164 | 0.543 | 0.105 | 0.744 | **-0.628** | **0.012** | -0.385 | 0.306 |
| Light sleep % | REM % | **-0.535** | **0.033** | -0.322 | 0.308 | **-0.796** | **0.000** | **-0.783** | **0.013** |
| Deep sleep % | REM % | 0.050 | 0.854 | -0.140 | 0.665 | 0.229 | 0.413 | 0.483 | 0.188 |

Blue cells show negative correlation coefficients, and red cells show positive correlation coefficients. Bold font indicates *P*-values <0.05, and bold with underlined font indicates *P*-values <0.01.

AHI, apnoea-hypopnea index; BMI, body mass index; REM, rapid eye movement; SAS, sleep apnoea syndrome.
